# Supplementary material for: Chromosome-level genome assemblies of 2 hemichordates provide new insights into deuterostome origin and chromosome evolution
Source: PLoS Biol. 2024 Jun 3;22(6):e3002661. doi: 10.1371/journal.pbio.3002661 (PMC11175523; doi:10.1371/journal.pbio.3002661)
Supplement: Attachment — Submitted filename: Response to Reviewers.docx [file pbio.3002661.s035.docx]

**Chromosome-level genome assemblies of two hemichordates provide new insights into deuterostome origin and chromosome evolution (PBIOLOGY-D-24-00409R1)** 

REVIEWERS' COMMENTS:


Reviewer #1:

This is an extraordinary paper that does much to resolve a variety of important open questions, provides exciting new insight, is sure to be a touchstone for many other projects in years to come. It is rigorously executed and well presented.

My comments address a few presentation issues. My relatively short review reflects how well structured and compelling the manuscript is in its current form.


line 108 - give a better indication when introducing these species of how closely related they are within Hemichordata. For example, do they bracket the ancestral node of the group? Their phylogenetic proximity has a big impact on interpreting the comparative results.

- We have added descriptions about relationships of the two species within Hemichordata.

“Hemichordates comprise two groups, the solitary enteropneusts and the colonial pterobranchs. In this study, we generated chromosome-level genome assemblies for two enteropneusts, the ptychoderid *Ptychodera flava* and spengelid *Schizocardium californicum*. Phylogenomic data showed that Ptychoderidae and Spengelidae are sister groups, together with Harrimaniidae constituting Enteropneusta (Cannon et al., 2014; Tagawa, 2016).”

line 349 - 346 - This part is confusing. It is odd to have bayesian phylogenetic support for deuterostomes in analysis of a dataset that does not have a synapomorphy for the group. Consistent with this, the posterior support values in Fig. 3D are quite low. I encourage the authors to clarify the presentations of this result - it is one of the questions of widest interest that is addressed here.

- The category data we coded include both intact and rearrangement events of corresponding chromosomes. Although the deuterostome species we investigated have no synapomorphy of fusion events, they shared nine unchanged ALGs. Thus, the Bayesian phylogenetic analysis support the deuterostome grouping. The low Bayesian probability support may be due to a small number of traits used in our study. To clarify this point, we modified the sentences into the following:

“We coded chromosomal status into category data, which was then converted into a binary matrix (Fig. 3b and c). Bayesian phylogenetic and clustering analyses based on these synteny-based characters united the deuterostomes as a clade to the exclusion of other animals (Fig. 3d-e). Notably, all the five deuterostome species we analyzed retain nine one-to-one matching chromosomes corresponding to the ancestral deuterostome state, however, no common chromosomal fusion (i.e., syntenic synapomorphy^17^) was identified.”

line 359 - missing an "and"

- We have added the missing “and”.

line 363-367 - wording is confusing here. Presumably they do share some rearrangement events that preceded their most recent common ancestor. Reword.

- We have reworded the sentence into:

“Therefore, the five major extant animal groups (i.e., ambulacrarians, chordates, spiralians, ecdysozoans and cnidarians) do not share common derived traits in terms of inter-chromosomal rearrangement events, and the observed chromosomal fusion events appear to be lineage-specific and have occurred before the diversification of each of these major animal groups.”

line 422 - reword. Current wording makes its sound like the dispersal is not retained.

- We have reworded the sentence into:

“All chordates appear to share a dispersal of deuterostome/bilaterian ALG R, but this ALG is retained as individual chromosomes in ambulacrarians (e.g., POC12 and SPU3) (Fig. 2b-c). Intriguingly, we found that POC12 and SPU3 are enriched for genes involved in DNA integration, including several transposase genes (Fig. 4e and Supplementary Fig. 17a and 18a).”

line 436 - reword to make it clear whether the hox cluster or chromosome is devoid of transposable elements

- We have reworded the subtitle into:

“Hox clusters in rearranged chromosomes”


Reviewer #2:
[identifies herself as Billie J Swalla]

This paper discusses data comparing two hemichordate Enteropneusta (worm-like) genomes, and the importance of these results to chordate origins, although the "new insights" referenced in the title are only new when key references are left out, as is done in this manuscript. Publication is not acceptable unless the early studies that suggested these results are added to the manuscript and references.

This manuscript shows that chromosomal assembly of two enteropneust hemichordate genomes show remarkable synteny - each hemichordate species has 23 chromosomes and synteny analyses with other phyla reveal that the Deuterostome ancestor was likely to have 24 chromosomes. This data is novel and interesting, and worthy of being published, but the background and discussion of ideas of chordate origins leaves out many key contributions. General discussions are presented and specific comments are written out below.

The authors seemed to have missed key early phylogenies that described the Deuterostomes in the context of chordate origins and the ancestor of the Deuterstomes hypothesized to contain gill slits (Cameron et al 2000). Please add this reference to the first paragraph in the Introduction, as the results were inferred from the Deuterostome phylogeny that showed echinoderms and hemichordates as sister groups. This result was disputed at the time, but genomic evidence has continued to agree with this result.

- We apologize for omitting the important publication by Cameron et al., 2000. We have added the reference in the first paragraph of the Introduction.

Line 374-375. "This fusion event therefore appears to be specific to ambulacrarians and does not provide evidence supporting either hypothesis." This is a very disappointing and misleading statement, the authors don't seem to believe their own results! If the Xenacoelomorpha do not share the ambulacrarian-specific chromosomal fusion, then it suggests that they are not ambulacraria, or deuterostomes.

- We thank the reviewer for this comment. We meant to say that the Xenambulacrarian hypothesis is not supported by our data, although we cannot rule out this hypothesis completely. We have modified the sentences as following:

"This fusion event therefore appears to be specific to ambulacrarians and does not provide evidence supporting the Xenambulacrarian hypothesis. However, the Xenambulacrarian hypothesis could not be ruled out by the current data, as the fusion could have occurred in the ambulacrarian lineage after Ambulacraria diverged from Xenacoelomorpha."

Specific Comments:
1.	Abstract and Introduction - "Deuterostomes are a "superphylum". Modern phylogenetics no longer refer to the old Linnaeus way of classifying animals. 
Deuterostomes are a monophyletic group of animals" is a much better way of describing this group of animals.

- We thank the reviewer for this comment. We have modified the sentences in Abstract and Introduction as suggested.

2.	Figure 1 Legend - Ptychodera flava (PFL) and Schizocardium californicum (SCA) should be switched in the Figure legend to match their position in the Figure: Branchiostoma floridae (BFL), Schizocardium californicum (SCA), Ptychodera flava (PFL), and Strongylocentrotus purpuratus (SPU).

- We have modified the figure legend as suggested.

3.	The colors in the little phylogeny to the left on Figure 1 are confusing, as they do not correspond to the colors on the right. It would be better to leave them black.

- We have changed the text colors to black (Figure 1a and b).

References:
Cameron CB, Garey JR, Swalla BJ. 2000. Evolution of the chordate body plan: New insights from phylogenetic analyses of deuterostome phyla. Proc. Natl. Acad. Sci. 97: 4469-4474.


Reviewer #3:

Lin and collaborators present here the results of a study that generated chromosome-level genome assemblies for two hemichordate species: Ptychodera flava and Schizocardium californicum.  The authors used comparative genomic approaches to infer the chromosomal architecture of the deuterostome common ancestor and delineate lineage-specific chromosomal modifications.  They found that hemichordate chromosomes exhibit remarkable chromosome-scale macrosynteny when compared to other deuterostomes, and can be derived from 24 deuterostome ancestral linkage groups.  The study also identified lineage-specific chromosomal fusion events and analysed the potential biological consequences of these rearrangements.  Additionally, the authors investigated the evolutionary history of the pharyngeal gene cluster and the distribution of transposable elements within Hox clusters.  Overall, the study provides very interesting insights into the evolution of deuterostome genomes and produce some
new hypothesis generating ideas: on the "posterior Hox flexibility" depending on transposable elements; and the pharynx having evolving by linkage of pre-existing bilaterian microsyntenic blocks on the deuterostome stem followed by lineage-specific changes.

The manuscript is very well written and structured, and the methods are sounds and properly justified.

I only have minor questions, mainly of it out of curiosity:

1. The GO enrichment analysis on heavily rearranged chromosomes is very interesting. However, do the authors know if this enrichment differs from that observed in other chromosomes? In other words, is it specifically different rather than just stochastically different? Additionally, are these GO terms exclusive to the heavily rearranged chromosomes within each species, or are they also found in the conserved chromosomes?

- We have performed GO enrichment analysis on the 9 conserved chromosomes. We discovered that GO terms which are enriched on the heavily rearranged chromosomes (such as DNA integration, germ layer formation, and axial patterning) are NOT observed on the conserved chromosomes in any of the species we examined. Additionally, the sea urchin-specific enriched GO terms, such as bone/otolith development in SPU1, were not found in other conserved sea urchin chromosomes. This finding indicates that the enriched GO terms on heavily rearranged chromosomes are likely to be specifically different rather than merely randomly different. We also identified some conserved chromosome-specific enriched GO terms, such as lipid transport in ALG L and lipid biosynthetic process in ALG M (shown in the figure below). We thank the reviewer for the suggestion to do this analysis. It would be interesting to further explore the differences in enriched GO terms between conserved and highly variable chromosomes in future studies.

2. In the GO enrichment experiment, do the authors have any way to correct for the differing (larger vs smaller) number of genes in each chromosome? Is a larger number of genes, for example, related to more GO terms? If so, how should we interpret the results here?

- We thank the reviewer for raising these questions. We have conducted a correlation analysis between the number of genes and the number of enriched GO terms on sea urchin (SPU), sea star (POC) and hemichordate (PFL) chromosomes. In general, the absolute values of Pearson correlation were relatively small (see below). Chromosomes with a larger number of genes do not always have more enriched GO terms. One exception is the sea urchin dataset, in which the Pearson correlation value was 0.78 between the number of genes and the number of enriched biological process GO terms, and 0.76 between the number of genes and the number of all enriched GO terms. However, the higher correlation is primarily due to the largest chromosome (SPU01), which has a much higher number of enriched GO terms compared to the other chromosomes we examined. To mitigate the effect of this outlier on the Pearson correlation, we calculated Spearman's rank correlation. The results showed negligible correlation (0.02 and -0.03) between the number of genes and the number of enriched GO terms based on the ranking of the numbers. Thus, our results indicate that a larger number of genes is not related to more enriched GO terms.

3. The authors mention that lineage-specific changes in the pharyngeal cluster might have contributed to the diversity of pharyngeal structures in deuterostomes. Are they referring to the gain/loss of specific genes within the cluster in different species? If so, could the authors add this information to the manuscript, complementing Figure 6?

- We indeed observed lineage-specific changes of the pharyngeal gene clusters. For example, we found *cnga* was duplicated in the hemichordate PFL, and the pharyngeal cluster of the sea urchin SPU is broken into three parts. However, the relationship between these genomic changes and the morphological evolution of pharyngeal structures remains unclear.

4. Typo in Figure 2: Change "chrodate" to "chordate."

- We have corrected the typo. Thank you very much.
